# Dynamic Progression of White Matter Hyperintensities in Alzheimer’s Disease and Normal Aging: Results from the Sunnybrook Dementia Study

**DOI:** 10.3389/fnagi.2016.00062

**Published:** 2016-03-24

**Authors:** Joel Ramirez, Alicia A. McNeely, Courtney Berezuk, Fuqiang Gao, Sandra E. Black

**Affiliations:** ^1^LC Campbell Cognitive Neurology Research Unit, Hurvitz Brain Sciences Research Program, Sunnybrook Research InstituteToronto, ON, Canada; ^2^Heart and Stroke Foundation Canadian Partnership for Stroke Recovery, Sunnybrook Health Sciences CentreToronto, ON, Canada; ^3^Graduate Department of Psychological Clinical Science, University of Toronto ScarboroughToronto, ON, Canada; ^4^Faculty of Medicine, School of Graduate Studies, University of TorontoToronto, ON, Canada; ^5^Department of Medicine, Neurology, University of Toronto and Sunnybrook Health Sciences CentreToronto, ON, Canada

**Keywords:** Alzheimer’s disease, white matter hyperintensities, small vessel disease, aging, dementia, longitudinal progression

## Abstract

Although white matter hyperintensities (WMH), markers of cerebral small vessel disease (SVD), are believed to generally increase over time, some studies have shown sharp decreases after therapeutic intervention, suggesting that WMH progression may be more dynamic than previously thought. Our primary goal was to examine dynamic progression of WMH in a real-world sample of Alzheimer’s disease (AD) patients and normal elderly (NC), with varying degrees of SVD. WMH volumes from serial magnetic resonance imaging (MRI; mean = 1.8 years) were measured from NC (*n* = 44) and AD patients (*n* = 113) with high and low SVD burden. Dynamic progression for each individual was measured using spatial overlap images to assess shrinkage, growth, and stable WMH volumes. Significant group differences were found for shrinkage (*p* < 0.001), growth (*p* < 0.001) and stable (*p* < 0.001) WMH, where the AD high SVD group showed the largest changes relative to low SVD and NC. Our results suggest spatial progression measured at the individual patient level may be more sensitive to the dynamic nature of WMH.

## Introduction

Recent advances in neuroimaging techniques have allowed for the volumetric quantification of white matter hyperintensities (WMH) of presumed vascular origin, a radiological biomarker of cerebral small vessel disease (SVD; Wardlaw et al., 2013) commonly visualized on T2-weighted (T2), proton density (PD), and fluid-attenuated inversion recovery (FLAIR) magnetic resonance imaging (MRI; Pantoni, 2010). The longitudinal progression of WMH have been previously associated with vascular risk factors such as hypertension (Wolfson et al., 2013) and diabetes (Taylor et al., 2003), aging (Vannorsdall et al., 2009), decreased gait performance (Silbert et al., 2008), cognitive decline (Longstreth et al., 2005; Kramer et al., 2007; Debette et al., 2011), and brain atrophy (Schmidt et al., 2005), as well as Alzheimer’s disease (AD) pathology at post-mortem (Erten-Lyons et al., 2013).

While it is generally accepted that WMH typically increase with aging and cerebrovascular disease progression, the estimated growth rates often vary between studies. Among the progression studies with quantitative WMH volumetrics producing continuous measures, baseline to follow-up imaging show increased WMH mean/median volumes ranging from 0.74 cc (~4 years) to 4.6 cc (~3.6 years) in normal elderly (NC) cohorts (Kloppenborg et al., 2014).

However, there is some recent evidence to suggest that in some cases, WMH burden may appear stable, with some studies even demonstrating regression or decreased WMH volume over time. Although early reports of this phenomenon were attributed to measurement error (Schmidt et al., 2003; Sachdev et al., 2007), recent radiological case reports have shown WMH regression in patients after cerebral infarction and ischemic stroke (Moriya et al., 2009; Durand-Birchenall et al., 2012; Cho et al., 2015), liver transplantation and improved hepatic encephalopathy treatment (Mínguez et al., 2007; Rovira et al., 2007), and carotid artery stenting (Yamada et al., 2010). Moreover, there is some evidence to suggest that drug treatments may decrease or slow the progression of WMH (Mok et al., 2009), leading some clinical trials to implement WMH burden as a neuroimaging outcome measure (Dufouil et al., 2005; White et al., 2013).

These previous findings suggest that WMH progression may be more dynamic than previously understood. In this study, we implement an imaging-based method to quantify the dynamic progression of WMH which allows for the spatial estimation of shrinking, growing, and stable WMH, at the individual patient level. We examined dynamic progression of WMH on a sample of NC participants (*n* = 44), and a real-world sample of AD patients (*n* = 113) with high and low WMH burden (Ramirez et al., 2015). Additionally, we contrasted these results with those obtained using the more conventional approach of just comparing baseline to follow-up net volumes.

## Materials and Methods

### Participants

Participants were sampled from the Sunnybrook Dementia Study (ClinicalTrials.gov NCT 01800214), an ongoing longitudinal study conducted at the Linda C. Campbell Cognitive Neurology Research Unit, Hurvitz Brain Sciences Program at the Sunnybrook Research Institute, and the University of Toronto, Canada. NC participants (*n* = 44) met strict criteria that included pre-screening and neuropsychological test performance that was within normal limits for their age and education levels. All AD participants (*n* = 113) met National Institute of Neurological and Communicative Disorders and Stroke—AD and Related Disorders Association criteria (McKhann et al., 2011) probable/possible AD. Probable AD included patients with none to mild WMH burden (*AD low SVD*, WMH < 3.5 cc: *n* = 56), and possible AD included patients with moderate to severe WMH burden (*AD high SVD*, WMH > 3.5 cc: *n* = 57). Although our *AD high SVD* group included patients with severe WMH burden, this sample had no evidence of any additional clinical criteria such as stroke temporally related to cognitive impairment, multiple or extensive infarcts, features of dementia with Lewy bodies, or other significant co-morbid disease that could substantially affect cognition (McKhann et al., 2011). Exclusion criteria included neurological diseases other than dementia, history of significant head trauma, tumors, normal pressure hydrocephalus, psychotic disorders unrelated to dementia, psychoactive substance abuse, and major depression.

All participants underwent a standardized comprehensive clinical evaluation and consented to neuropsychological testing, standardized brain MRI, and consented to the use of their data for research purposes. Research was ethically conducted and approved by the Sunnybrook Research Ethics Board.

### MRI Acquisition

All brain imaging was acquired on a 1.5T GE Signa system (Milwaukee, WI, USA) with the following protocol: a T1-weighted axial 3D spoiled gradient recalled (SPGR): Echo Time (TE) = 5 ms, Repetition Time (TR) = 35 ms, Number of Averages (NEX) = 1, flip angle = 35°, Field Of View (FOV) = 22 cm, in-plane voxel size = 0.86 × 0.86 mm, slice thickness range: 1.2–1.4 mm and an interleaved PD/T2-weighted axial dual-echo spin echo: TE = 30/80 ms, TR = 3000 ms, NEX = 0.5, flip angle = 90°, FOV = 20 cm, in plane voxel size = 0.78 × 0.78 mm, slice thickness = 3 mm. The mean interscan interval (ISI) between baseline and follow-up scan was approximately 2 years (Table 1).

**Table 1:** **Demographics and raw volumetrics for NC, *AD low SVD* and *AD high SVD* groups**.

	NC (*n* = 44)	*AD low SVD* (*n* = 56)	*AD high SVD*	*P* (*n* = 57)
**Demographics**
Age, years (Baseline)	69.4 (7.0)	67.9 (8.0)	74.3 (8.3)	***
Sex, n (%) male	19.0 (43.2)	28.0 (50.0)	22.0 (38.6)	ns
Education, years	15.7 (2.7)	13.6 (3.5)	12.9 (3.5)	***
Interscan Interval, years	2.0 (1.2)	1.8 (1.1)	1.7 (0.8)	ns
MMSE/30 (Baseline)	28.9 (1.3)	23.9 (3.3)	22.8 (4.3)	***
MMSE/30 (Follow-up)	28.6 (1.2)	21.4 (5.8)	20.3 (6.2)	***
**Baseline volumes**
TIC	1216.6 (126.8)	1189.3 (128.4)	1191.7 (143.5)	ns
BPF, % (Baseline)	79.2 (3.5)	74.4 (4.5)	73.7 (3.6)	***
WMH (Baseline)	4.4 (5.6)	1.3 (0.9)	14.0 (13.7)	-
WMH (Follow-up)	5.1 (6.7)	1.7 (1.3)	15.7 (13.5)	-
**Volume change**
**(Follow-up - Baseline)**
WMH difference	0.8 (2.0)	0.3 (1.1)	1.7 (5.1)	ns
vCSF difference	2.5 (2.2)	7.2 (7.0)	8.4 (7.2)	***
**Dynamic volume**
**change (Spatial)**
WMH shrink	1.3 (1.2)	0.7 (0.5)	3.8 (3.3)	***
WMH grow	2.1 (2.3)	1.1 (0.9)	6.3 (4.3)	***
WMH stable	−0.6 (21.6)	0.5 (0.4)	9.3 (11.0)	***

Data are presented as Mean (SD). Raw volumes are presented for transparency, statistical analyses were performed on normalized data. Volumetrics are reported in cubic centimeters (cc). ***p < 0.001. NC, normal control; AD, Alzheimer’s disease; SVD, small vessel disease; MMSE, mini mental state exam; TIC, total intracranial volume; BPF, brain parenchymal fraction; WMH, white matter hyperintensity; vCSF, ventricular cerebrospinal fluid.

### Tissue Quantification

Tissue quantification was accomplished using in-house segmentation tools (www.sabre.brainlab.ca) that yielded an individualized volumetric profile of brain tissue and volumetric imaging markers of SVD. Baseline and follow-up volumes for gray matter (GM), white matter (WM), sulcal cerebrospinal fluid (sCSF), and ventricular CSF (vCSF) were obtained using a local histogram-based automatic segmentation tool (Kovacevic et al., 2002). Baseline and follow-up volumes for SVD markers, such as WMH and cystic fluid-filled lacunar-like infarcts or “black holes” (BH), were obtained using a semi-automatic tri-feature (T1/PD/T2) segmentation algorithm called Lesion Explorer (Ramirez et al., 2011, 2014). Baseline head size volumes were measured by the total intracranial capacity (TIC), which included all supratentorial brain matter and CSF voxels. Baseline global brain atrophy was assessed by the Brain Parenchymal Fraction (BPF) which was calculated on an individual basis as follows: BPF = (GM + WM + WMH)/TIC.

### WMH Progression Volumetrics

Volumetric data was generated using the conventional method by calculating the net change in total WMH volume from baseline to follow-up (total volume change = follow-up − baseline) for comparison with our dynamic progression method.

For our dynamic progression method, we used spatial location to gain more information about the potential dynamic nature of WMH. Baseline and follow-up T1 MRIs were transformed into each individual’s intermediate space using linear registration (FSL’s “FLIRT”, 6° of freedom; Jenkinson et al., 2012). This was accomplished by registering each baseline T1 image to follow-up using “FLIRT” and then using the “avscale” command to generate forward and backward halfway transformations. This half transformation was then applied to each baseline and follow-up image to create an intermediate image for both time points, which was then averaged to create one image that represented both baseline and follow-up in an intermediate space.

**Figure 1:**
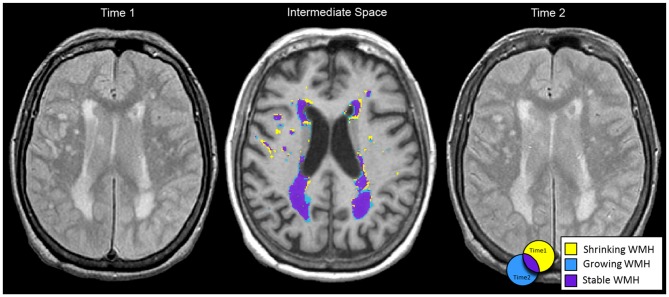
**Baseline (left) and follow-up (right) MRIs were half transformed into intermediate space (middle) using linear registration (FLIRT).** Shrinking (yellow), growing (blue) and stable (purple) white matter hyperintensities (WMH) spatial segmentations are displayed in intermediate space for a 71 year old female with Alzheimer’s disease (AD). Net change in WMH total volume from baseline to follow-up was −1.5 cc. However, when considering dynamic progression based on spatial information: shrinking WMH = −8.2 cc, growing WMH = +6.3 cc, and stable WMH = 32.4 cc.

**Figure 2:**
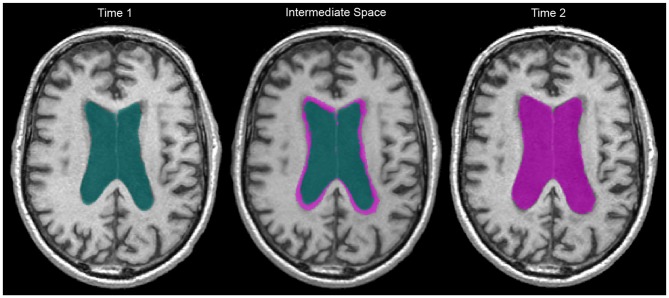
**Two year ventricular expansion in a 60 year old man living with AD.** Baseline vCSF = 83.6 cc, follow-up vCSF = 119.0 cc. Green indicates baseline vCSF voxels, pink indicates follow-up (right) and growth (middle). WMH within vCSF growth regions were subsequently removed to account for ventricular expansion.

Once the intermediate T1 was generated, we registered WMH and vCSF segmentations for baseline and follow-up to each individual’s intermediate space. We then used “fslmaths” to create spatial difference/overlap masks between baseline and follow-up. Spatial change in WMH included: shrinking (WMH present in baseline but not in follow-up), growing (WMH not present in baseline but present in follow-up), as well as stable WMH (present in both baseline and follow-up; Figure 1). Additionally, WMH voxels that fell within the ventricles were masked out to account for ventricular expansion into pre-existing WMH voxels (Figure 2).

### Statistical Analysis

Statistical analyses were performed on a PC using IBM SPSS Statistics (version 22). AD patients were sub-divided into *AD low SVD* (*n* = 56) and *AD high SVD* (*n* = 57) groups based on the median split of WMH at baseline (Behl et al., 2007; McNeely et al., 2015). Multivariate analysis of covariance (MANCOVA) were performed to examine potential differences in basic demographics and baseline characteristics (age, sex, education, ISI, Mini Mental State Exam (MMSE), TIC, vCSF difference and BH difference), secondary variables of interest (BPF, GM difference and WM difference), and the primary between group analyses for dynamic progression of WMH (i.e., shrink, grow, stable, and total volume). Bonferroni corrected pairwise comparisons were conducted *post hoc*. Due to the commonly observed skewed distribution of SVD imaging markers (DeCarli et al., 2005; Barnes et al., 2013), WMH change volumes were log transformed to allow for parametric testing. Baseline TIC was used to account for variations in head size, baseline vCSF and BPF were included to account for brain atrophy, and baseline MMSE (Folstein et al., 1975) scores were included to account for disease severity.

## Results

**Figure 3:**
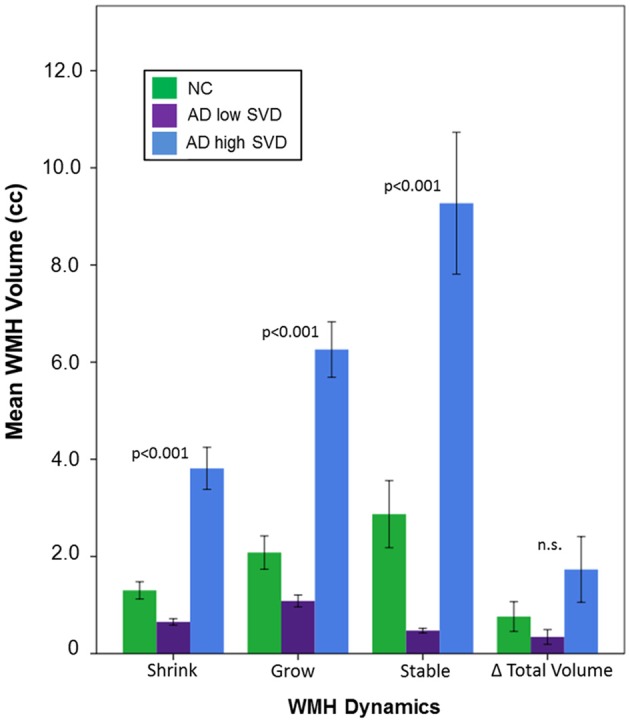
**Dynamic changes in WMH volume over a mean interscan interval (ISI) of 2 years for NC, AD low small vessel disease (SVD), and AD high SVD groups.** *P* values represent significant differences between all diagnostic groups, based on estimated marginal means, accounting for baseline age, years of education, mini mental state exam (MMSE), and vCSF.

Demographic and volumetric data are summarized on Table 1. Age (*p* < 0.001), education (*p* < 0.001), and MMSE at baseline and follow-up (*p* < 0.001) were found to be significantly different between groups. Head-size (TIC), sex, and ISI were not significantly different between groups and were subsequently removed as covariates in further analyses. All subsequent analyses were adjusted for baseline age, education, disease severity, atrophy, and incidental infarcts. Significant between group differences were demonstrated in baseline BPF measures (*p* < 0.001), GM change (*p* < 0.001), WM change (*p* < 0.001), and vCSF change (*p* < 0.001). Interestingly, the conventional method yielded baseline to follow-up total net WMH volume changes that were not significantly different between groups (Table 1: “Volume change”, Figure 3: “Δ Total Volume”).

However, significant between group differences were demonstrated using dynamic progression of WMH metrics: WMH shrinkage (*p* < 0.001), WMH growth (*p* < 0.001), and stable WMH (*p* < 0.001). Bonferroni corrected *post hoc* tests revealed that dynamic progression of WMH was significantly different between all groups, where the *AD high SVD* group had significantly more shrinkage, growth, and stable WMH volumes than NC (all, *p* < 0.001) and *AD low SVD* groups (all, *p* < 0.001; Figure 3).

## Discussion

Our main findings suggest that WMH progression may be more dynamic than previously thought. Specifically, AD patients with significant radiological signs of cerebral SVD pathology at baseline exhibited more dynamic progression of WMH over a 2 year period, when compared to AD patients with minimal SVD burden and their NC counterparts.

Progression rates of WMH volumes from serial MRI studies vary considerably, however, the overall net volume change results in our study were within the range of values previously reported in the literature (see Table 2; Garde et al., 2005; Schmidt et al., 2005; Kramer et al., 2007; Silbert et al., 2008; Marquine et al., 2010; Wolfson et al., 2013). Interestingly, the largest net volume increase of WMH in our study was observed in AD patients with high SVD burden at baseline, which likely reflects previous reports that demonstrate how baseline WMH burden can significantly predict higher progression rates (Prins and Scheltens, 2015).

**Table 2:** **Summary of serial MRI studies examining WMH change over time**.

					Volume		
Publication	*n*	Study	Baseline age (y)	Sample	Baseline	Follow-up	Difference*	Mean ISI
**Current study**
Ramirez et al. (2015)	44	Sunnybrook Dementia Study, Canada	69.4 ± 7.0	Normal elderly controls	4.4 (5.6)	5.1 (6.7)	+0.8 (2.0)	2.0 y
Ramirez et al. (2015)	56	Sunnybrook Dementia Study, Canada	67.9 ± 8.0	AD patients with low SVD burden	1.3 (0.9)	1.7 (1.3)	+0.3 (1.1)	1.8 y
Ramirez et al. (2015)	57	Sunnybrook Dementia Study, Canada	74.3 ± 8.3	AD patients with high SVD burden	14.0 (13.7)	15.7 (13.5)	+1.7 (5.1)	1.7 y
**Population studies**
Wolfson et al. (2013)	67	University of Connecticut Health Center, USA	81.7 ± 3.9	Community dwelling elderly	1.0	1.7	+0.7	4.0 y
Garde et al. (2005)	26	Danish Glostrup 1914 cohort	80.7 ± 0.4	Cohort study	4.7	9.3	+4.6	3.6 y
Marquine et al. (2010)	110	Duke University Medical Center, USA	70.7 ± 5.6	Normal elderly controls	4.9	6.1	+1.2	2.0 y
Kramer et al. (2007)	50	Multi-center California, USA	73.9 y ± 6.6	Healthy elderly controls	8.0	10.4	+2.4	3.7 y
Schmidt et al. (2005)	292	Austrian Stroke Prevention Study (3 y)	60.2 ± 6.3	Community dwelling elderly	1.3	1.92	+0.6	3.0 y
Schmidt et al. (2005)	243	Austrian Stroke Prevention Study (6 y)	60.2 ± 6.3	Community dwelling elderly	1.4	2.7	+1.4	6.0 y
Cho et al. (2015)	100	Seoul St. Mary’s Hospital, Korea (progress, *n* = 32)	67.5 ± 11.8	Ischemic stroke patients	2.8^†^	6.1^†^	+3.3	2.8 y
Cho et al. (2015)	100	Seoul St. Mary’s Hospital, Korea (regress, *n* = 14)	67.5 ± 11.8	Ischemic stroke patients	2.8^†^	0.9^†^	−1.9	2.1 y
Silbert et al. (2008)	104	Oregon Brain Aging Study	85.1 ± 5.6	Healthy community wilunteers	9.7	14.1	+4.4	9.1 y
**Case reports**
Rovira et al. (2007)	27	Pre-post liver transplant	55	Cirrhosis patients	1.3^†^	0.7^†^	−0.6	9 m
Durand-Birchenall et al. (2012)	1	Post secondary stroke prevention	69	Lacunar stroke	9.2	4.5	−4.7	6 m
Mínguez et al. (2007)	1	Pre-post intervention (neomycin)	70	Hepatic encepalopathy (hep C)	7.5	2.0	−5.5	6 m
Mínguez et al. (2007)	1	Pre-post intervention (neomycin)	65	Hepatic encepalopathy (alcoholic cirrhosis)	25.9	16.6	−9.3	1 m
Mínguez et al. (2007)	1	Pre-post intervention (neomycin)	73	Hepatic encepalopathy (hep C cirrhosis)	18.6	10.4	−8.2	6 m

*Estimated change based on reported means at baseline and follow-up. ^†^Median. Abbreviations: ISI, interscan interval; y, years; m, months.

Despite these reports of overall net increases of WMH volume over time, some studies have demonstrated differential progression rates spatially across various brain regions, and even regression or decreases, providing early support for the potential dynamic nature of WMH. When the spatial distribution of WMH have been considered, differential progression rates have been shown in the periventricular compared to deep white brain regions (van den Heuvel et al., 2004; Sabayan et al., 2015). Additionally, significant WMH regression and signal attenuation has also been previously demonstrated in radiological case reports with pre- and post-therapeutic intervention imaging (Mínguez et al., 2007; Rovira et al., 2007; Durand-Birchenall et al., 2012).

Moreover, a recent serial MRI study performed on ischemic stroke patients demonstrated both progression *and* regression, with WMH regression observed in 21.5% of stroke patients (Cho et al., 2015). These findings are particularly important in individual cases where there are signs of simultaneous progression (growing), regression (shrinking), and stable WMH, as demonstrated in our study. The loss of information from baseline to follow-up from volumetric averaging can further be reflected in our group analysis, where the conventional WMH net volume change between groups was non-significant but dynamic progression rates were.

In light of the commonly accepted view that the pathogenesis and clinical characteristics of “WMH of presumed vascular origin” can be attributed to a set of heterogeneous and complex etiologies associated with cerebral SVD (Gouw et al., 2011), our findings provide further support for the dynamic nature of the underlying pathology that is reflected by these radiological entities. Although poorly understood, there is some evidence to suggest that a common substrate of diffuse WMH in the periventricular regions of the brain may reflect a form of venous stenosis (Moody et al., 1995; Black et al., 2009; Pantoni, 2010). Early findings from imaging-pathology case studies suggest that the confluent periventricular WMH may reflect edema related to collagenosis of the periventricular deep medullary veins (Gao et al., 2014).

Fluid from chronic edema resulting from increased blood-brain barrier permeability (Farrall and Wardlaw, 2009), intraparenchymal venous abnormalities (Brown et al., 2009), and/or disturbances in interstitial fluid circulation (Weller et al., 2009), may also explain the dynamic shrinkage and growth of WMH that was demonstrated in our study, as edematous fluid has been known to partially resolve after time has elapsed (or therapeutic intervention is performed). Thus, edema visualized as WMH on MRI may be an indicator of complex multiple underlying pathologies that include venous collagenosis and/or arteriolosclerosis. These chronic SVD pathologies may result in increased blood-brain barrier leakage and interstitial fluid accumulation due to increased venous pressure and decreased fluid drainage along the perivascular channels as a consequence of vascular rigidity and reduced vascular pulsation. These proposed multi-factorial etiologies make it challenging to attribute regression to “recent” vs. “older” lesions. However, collagenosis and/or arteriolosclerosis are insidious chronic processes, which could gradually lead to increased fluid accumulation, but also allows time for fluid resolution, where WMH regression may reflect the rate of resolution (clearance or drainage of fluid) when it is temporarily greater than the rate of fluid accumulation. Moreover, this dynamic process of fluid accumulation and clearance may also be modulated by other factors, such as blood pressure (Hauser et al., 1988), during image acquisition.

Additionally, there is some evidence to suggest that a proportion of focal infarcts may resemble and/or distort WMH volumes due to their similarity in signal intensity (Wang et al., 2012). As incomplete and partially cavitated lacunar infarcts and acute small subcortical strokes tend to resemble WMH, when measured longitudinally, many of these lesions (94%) have been shown to completely or incompletely cavitate, reduce in diameter, and/or disappear (5%) on follow-up imaging (Lammie et al., 1998; Potter et al., 2010; Loos et al., 2012). This process may also partially account for the dynamic progression findings in our study.

One limitation of our study is the lack of additional time points for neuroimaging measures. As the Sunnybrook Dementia Study is a longitudinal observational study, additional follow-up scans for each individual will become available for future analysis to provide a more accurate picture regarding the dynamic changes of WMH in a real-world sample of dementia patients with varying degrees of comorbid SVD pathology (Ramirez et al., 2015). Additionally, as both a benefit and a caveat, the ability of our method to spatially account for vCSF expansion is particularly relevant in studies on aging and dementia since ventricle size is an important indicator of brain atrophy, commonly observed in AD-related neurodegeneration and normal aging (Nestor et al., 2008; Apostolova et al., 2012; Madsen et al., 2015). Unfortunately, while this dynamic feature in our method accounts for ventricular expansion, it may also introduce potential error, as baseline WMH voxels which overlay onto newly labeled vCSF voxels are removed due to ventricular expansion at follow-up. Should the baseline volumes of periventricular WMH that eventually become engulfed into the ventricular compartment be quantified as a separate unique volume? To our knowledge, previous studies have not considered this, although many studies (including this one) include ventricular volume as a covariate to account for medial atrophy.

Future work examining dynamic progression with spatial information from both WMH and ventricular expansion would be beneficial when examining vascular co-pathology in AD and neurodegenerative populations. Future therapeutic strategies that target cerebrovascular contributions to AD dementia, such as the use of anti-hypertensive treatments (Dufouil et al., 2009; Jochemsen et al., 2012, 2015; White et al., 2013), may be particularly interested in the shrinkage, growth, and stable metrics examined in our study. Although several complex statistical modeling approaches have been proposed to analyze longitudinal structural data (McArdle et al., 2004; Grimm et al., 2012), our approach is relatively simple by comparison and can be implemented into any serial imaging study in parallel with the conventional baseline to follow-up net volume change approaches.

## Conclusion

The findings from this study suggest that individual WMH volume changes are more dynamic than previously thought. The simultaneous shrinkage and growth of WMH was particularly evident in AD patients with significant signs of cerebral SVD. Given that our dynamic progression measures provided information that was above and beyond the standard net volume change, our results suggest that the measurement of dynamic volumetrics for WMH may be particularly valuable in clinical trials that use imaging outcome measures and may further elucidate the complex pathogenesis and longitudinal progression of this radiological entity we commonly refer to as *WMH of presumed vascular origin*.

## Author Contributions

JR: literature review, manuscript writing, statistical analysis, project development. AAM: neuroimaging analysis, literature review, manuscript writing. CB: neuroimaging analysis, manuscript editing, statistical analysis. FG: project development, radiological consultation, manuscript editing. SEB: patient assessment, project development, principal investigator, manuscript writing.

## Conflict of Interest Statement

The authors declare that the research was conducted in the absence of any commercial or financial relationships that could be construed as a potential conflict of interest.
